# How does red culture affect corporates’ cash holdings? Evidence from China

**DOI:** 10.1016/j.heliyon.2023.e15435

**Published:** 2023-04-14

**Authors:** Zuohong Li, Xiaohui Chen, Bo Yang

**Affiliations:** a School of Economics, Sichuan University, Chengdu, China; b Faculty of Economics and Business Administration, Yibin University, Yibin, China

**Keywords:** Red culture, Corporates' cash holdings, Heterogeneity, Long-term investment, Investment opportunity, Duality

## Abstract

Cash holdings are an essential element of corporate decision-making. Culture, such as the unique Chinese red culture created by the Communist Party of China during the Revolutionary War, affected corporate economic decisions. This study systematically evaluated the impact of red culture on corporate cash holdings. Based on a theoretical analysis, this study conducts an empirical analysis using data on Chinese-listed companies from 2008 to 2021. The results show that: (1) red culture reduces corporate cash holdings. For each unit of red cultural influence, corporate cash holdings decreased by 1.5979% points. (2) The role of red culture in reducing corporate cash holdings is heterogeneous; it plays a greater role in high long-term investment corporations than in low long-term investment corporations, in high investment opportunity corporations than in low investment opportunity corporations, and in duality corporations than in non-duality corporations. This study can help global investors understand China's culture and capital markets and will be the first step toward exploring the economic impact of red culture.

## 1 Introduction

Since the beginning of the 21st century, the cash holdings of U.S. listed companies have increased [1,2]. The sharp rise in corporate cash holdings has become a global phenomenon over the past 30 years [3]. By contrast, the cash holdings of Chinese listed companies exhibit a continuous downward trend. The cash holdings of Chinese listed companies will decrease from 9.65% in 2008 to 6.84% in 2021, a decrease of 2.81% over 13 years. Why do Chinese listed companies’ cash holdings decline instead of rising?

Culture is an important factor affecting the production and operation of corporations. In China, red culture is an advanced culture with a rich revolutionary spirit created by the Communist Party of China during the Revolutionary War. It was a unique culture that the Chinese people gradually formed during the war for national independence. Its uniqueness lies in its solid, rich red core. The core of red culture lies in overcoming all difficulties in winning [4], integrating theory with practice [4,5], maintaining close ties with the masses [4,5], the spirit of revolutionary optimism [4,6], unswerving ideals and beliefs [7], fine quality of perseverance [8], loyalty, honesty, fairness, mutual love, and principles [9]. Hence, is red culture responsible for the continuous decline in cash holdings of Chinese listed companies? To help global investors better understand China's culture and capital markets. This study examined the impact of red culture on corporate cash holdings.

First, cash holdings are closely connected to managers [10,11]. To explore the influence of red culture on the cash holdings of Chinese listed companies, this study, based on the theory of psychological ownership, qualitatively assumes that red culture can improve managers’ psychological ownership. Psychological ownership is conducive to reducing corporate cash holdings. The aspects of red culture —seeking truth from facts, loyalty, honesty, fairness, hard work, mutual love, and mutual help [9]—are conducive to implementing ethical leadership and mobilizing employees' enthusiasm, which, in turn, is favorable for reducing corporate cash holdings. Accordingly, we hypothesize that red culture can reduce the cash holdings of listed Chinese companies. Based on the data of Chinese listed companies from 2008 to 2021, we empirically test the impact of red culture on corporate cash holdings using a time and individual two-way fixed-effects model.

Second, we attempted to measure the influence of red culture. However, there are insufficient studies on cultural influence. Considering religion as an important cultural aspect, Hilary and Hui (2009) [12] measured the influence of religion on the proportion of religious believers among the total regional population when studying the impact of religion on corporate risk-taking. In China, the red culture has been deeply integrated into the people's “cultural gene” and “spiritual blood”: all Chinese people have been affected by the red culture [5]. Therefore, this study collects all information on red culture enterprises (i.e., enterprises whose business scope includes red culture) from qcc.com, tallies them in each Chinese city from 2008 to 2021 according to the time of establishment and place of registration, and divides their total number by the total population of the city as the proxy variable for red culture influence. Red cultural enterprises conduct red cultural education and training, passing it on to successive generations. Therefore, the greater the number of red cultural enterprises in a city per unit population, the stronger the supply and demand for red cultural education, training, and inheritance. The greater the impact of red culture on other enterprises in the city. Based on this argument, we constructed independent variables for the empirical tests.

Finally, based on the psychological ownership of managers and the resulting ethical leadership, we take long-term investments, investment opportunities, and whether the board chairperson concurrently serves as a CEO as categorical variables. Based on the data of China's A-share listed enterprises from 2008 to 2021, we use the variable coefficient of the individual fixed effects model to test for heterogeneity (i.e., explore whether there is heterogeneity in the impact of red culture on corporate cash holdings).

Based on the above research design, we made the following conclusions: First, the greater the influence of red culture on a corporation, the lower its corporate cash holdings. Red culture's induced psychological ownership among managers is conducive to reducing corporate cash holdings. The conclusion that red culture reduces corporate cash holdings remains valid even after robustness checks, such as replacing dependent variables, changing the measurement method of the independent variables, eliminating endogeneity, and changing the estimation model. Second, the role of red culture in reducing corporate cash holdings is heterogeneous: its role in high long-term investment corporations is greater than in low long-term investment corporations, its role in high investment opportunity corporates is greater than in low investment opportunity corporates, and its role in CEO duality corporates is greater than in non-CEO duality corporates.

Our findings have several theoretical and practical implications for future research. First, we explain the decline in continuous cash holdings of Chinese listed companies, which remains unclear. Since the beginning of the 21st century, the cash holdings of U.S.-listed companies have continued to rise [13], while those of Chinese-listed enterprises have continued to decline. From a cultural perspective, this study concludes that red culture reduces corporate cash holdings, explaining this continuous decline. Second, our findings can be referenced by corporations outside China. While it is difficult for corporations outside China to benefit from red culture, they can take other measures to improve managers’ psychological ownership, encouraging them to implement ethical leadership.

On the theoretical front, our study contributes immensely to the literature. First, it contributes to the literature on corporate cash holdings. Since Keynes (1936) [14] creatively studied the three motives for corporate cash holdings—transaction, prevention, and investment —many other scholars have identified several factors that influence corporate cash holdings [11,13,15,16]. Internal factors such as financing constraints [13,16] and corporate governance [11] and external factors such as political corruption [16] and local financial development [15] affect corporate cash holdings. Therefore, this study enriches the literature on the factors that influence cash holdings by discussing the impact of red culture on corporate cash holdings.

Second, our study contributes to the emerging literature on the economic consequences of culture. Under different cultural backgrounds, people have different future expectations regarding their economic behavior and decision-making [10,17,18]. The existing literature usually distinguishes between collectivism and individualism to study their economic impact [17,19]. Andries and Balutel (2022) [17] studied the effects of national culture on systemic risk. However, research on the effect of red culture on corporate cash holdings and heterogeneity is lacking. Contrary to the existing practice of studying the impact of culture on the economy from the perspectives of collectivism and individualism, this study conducts an empirical test based on Chinese listed companies’ data from 2008 to 2021, focusing on the impact of red culture on corporate cash holdings, and enriching the literature on the impact of culture on the economy.

Finally, this study contributes to the literature on psychological ownership. Extensive research has been conducted on psychological ownership [[20], [21], [22]]. These studies have mainly focused on the impact of psychological ownership on employee happiness, effort, performance, and loyalty. For example, psychological ownership has a significant positive association with the subjective happiness of the employees [21]. Service providers with psychological ownership of the service setting enhance customers' behavioral loyalty [20]. Therefore, by considering the impact of psychological ownership of managers on cash holdings, this study confirms that psychological ownership can reduce corporates’ cash holdings, expands the research scope of psychological ownership, and enriches the literature related to the economic impact of psychological ownership.

The remainder of this paper is organized as follows: Section 2 provides a literature review and clarifies our testable hypothesis, Section 3 describes our empirical design, Section 4 discusses our main empirical results and robustness tests, Section 5 presents the heterogeneity analysis, and Section 6 summarizes the study.

## 2 Hypothesis development

### 2.1 Red culture and psychological ownership

Psychological ownership is a psychological state in which people think that the target is “their” [23]; that is, people's sense of possession of the target [24,25]. This sense of possession makes people regard the target as their extension [23] and affects their attitudes, motivations, and behaviors toward it [25]. Red culture can stimulate managers to possess psychological ownership toward corporations. Pierce, Kostova, and Dirks (2001) [23] believe that people's psychological ownership arises from three motives: the space sense of “home,” self-efficacy, and self-identity. Red culture can act on these three motives, stimulating managers to develop psychological ownership towards a corporation. The first aspect is enhancing the sense of belonging and producing the space sense of “home”. “Home” provides people with a comfortable, happy, and safe environment; thus, the space sense of “home” is one of the motives for people to produce psychological ownership [23,25]. Red culture has been deeply integrated into the Chinese people's cultural genes and spiritual blood [5]. Red culture can arouse positive emotions and enhance the sense of identity [26]. In the corporate environment, this sense of identity can be specifically transformed into a sense of identity to the corporate, resulting in the sense of belonging and a space sense of “home”. For example, through regular and irregular red culture learning and communication, managers can feel the care of shareholders and the board of directors, thus increasing their sense of belonging and creating a space sense of “home.” The second aspect is enhancing one's sense of achievement and deriving a sense of self-efficacy. Based on the possession of the target object, people obtain a sense of self-efficacy by gaining satisfaction, which is the second motivation to produce psychological ownership [23]. “All believe in the masses, and all rely on the masses” is an important content of red culture [4,5]. Specifically for corporates, “trust the masses and rely on the masses” means that the shareholders and board of directors will respect the initiative of managers and fully mobilize their enthusiasm. Managers are more likely to obtain a sense of achievement in their work based on their initiative and enthusiasm. This helps improve their sense of self-efficacy, resulting in the psychological ownership of the corporation. The third aspect involves increasing participation and promoting self-identity. People define, express themselves, and distinguish themselves from others through objects; hence, self-identity motivates psychological ownership [23,25]. In the corporate environment, the red culture of “believing in the masses and relying on the masses” [4,5] means that the shareholders and board of directors will listen more to managers' opinions, which is conducive to the participation of managers in corporate decision-making. This will urge managers to invest more ability and energy in the corporation to gradually integrate it, thus promoting their self-identity. In short, red culture is conducive to managers' psychological ownership towards corporations.

### 2.2 Red culture and ethical leadership

Brown, Treviño, and Harrison (2005) [27] propose a theory of ethical leadership based on social learning theory. According to this theory, based on the psychological processes of observation, learning, imitation, and identification, leaders can influence the ethical behavior of their subordinates, thus forming ethical leadership [27]. Ethical leaders exercise power in an altruistic way; care about the welfare of their subordinates [28]; show what normative and appropriate behavior is through personal actions and interpersonal interactions; and stimulate the ethical behavior of their subordinates through communication, reinforcement, and decision-making [27]. Existing studies have found that red culture contains noble ethical sentiments, is the soul of one's thoughts, and is the driving force of action; constructive criticism and leadership values influence ethical concepts [29].

Ethical values such as loyalty and honesty, fairness and decency, mutual love, and respect for principles— the pillars of red culture—improve people's virtues [9]. Managers' ethical conduct is essential for ethical leadership [30]. As red culture has been deeply integrated into the Chinese cultural gene and spiritual blood [5], its ethical sentiment can promote managers' ethical leadership. Red culture was established during the Chinese revolution, embodied in the quality of daring to sacrifice and the willingness to contribute, and the refined style of stressing principles and rejecting privileges [9]. At the corporate level, red culture can enable managers to exercise management power altruistically without paying attention to privileges, which is expected in ethical leadership [28].

### 2.3 Testable hypotheses

**Fig. 1:**
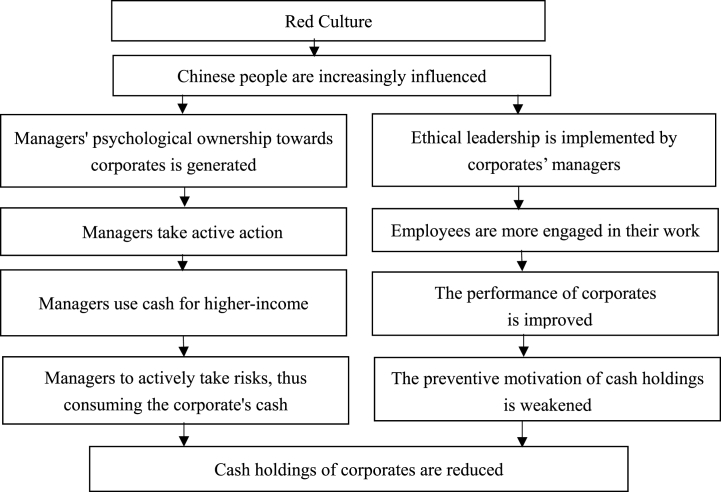
The impact path of red culture on cash holdings of corporates.

As previously discussed, red culture can help managers generate psychological ownership towards corporations and promote ethical leadership, which is conducive to reducing their cash holdings (Fig. 1).

First, managers' psychological ownership of a corporation reduces their cash holdings. According to possession psychology, psychological ownership produces a positive attitude toward a target and enhances the sense of responsibility [24,25]. When psychological ownership towards an organization is generated, individuals have a strong sense of organizational identity. They are proud of their organization, which prompts them to voluntarily invest more time and effort in the organization [31]. As cash holdings entail strong liquidity but low income, investing more time and effort to improve and protect, corporations allow managers to use cash for higher-income production and operational activities, thereby reducing cash holdings. Improving the sense of responsibility toward the target implies that psychological ownership stimulates positive organizational effects such as risk-taking behaviors [23]. Risk-taking is a resource-consuming activity that strongly depends on resources such as capital [32,33]. Enhancing the sense of responsibility encourages managers to actively take risks, thereby consuming corporate cash and reducing cash holdings.

Second, ethical leadership can reduce corporate cash holdings. Managers with ethical leadership exercise power altruistically and care about their subordinates [28]. The ethical leadership theory holds that leaders' excellent characteristics, such as honesty and caring for others, can produce positive effects, improve employees' job satisfaction [27,30], improve employees' well-being [30], and cultivate employees' risk spirit [27]. This can improve employees' workplace performance and enhance corporate profitability. Ethical managers focus on preparing subordinates for managerial positions, boosting their confidence in achieving collective goals, and encouraging them to think independently and question existing ways of doing things [28], which improves employees' enthusiasm and initiative in work and corporate profitability. Ethical managers focus on cultivating subordinates' creative and critical thinking skills, providing them with development opportunities, welcoming positive and negative feedback, recognizing their efforts, and sharing information with them. They also emphasize ethical standards based on the organization's and society's collective interests [28]. This encourages employees to focus on the corporation's interests, weakens their interests, and enhances their performance. Enhanced corporate performance strengthens the ability to obtain cash flows, thus weakening the preventive motivation for cash holdings.

In summary, red culture is conducive to cultivating managers' psychological ownership and encouraging them to actively implement ethical leadership, which reduces corporate cash holdings. Therefore, we propose the following hypotheses:

H1 Red culture can reduce corporates' cash holdings.

## 3 Empirical design

### 3.1 Cash holdings

Following the literature [[34], [35], [36]], we divide cash and cash equivalents by total assets to obtain *CS* as a proxy variable for corporate cash holdings. Florackis and Sainani (2018) [37] studied corporate cash holdings and made industry adjustments to them. Chinese scholars such as Yang, Wu, and Zeng (2015) [38], Zhang and Wu (2014) [39], and Zhou, Luo, and Xu (2020) [40] examined the cash holdings of Chinese listed companies. They subtracted the industry average from corporate cash holdings to adjust for the industry. In this study, following their methods, we subtract the industry average from *CS* to obtain *rCS* as a proxy variable for corporate cash holdings while checking robustness.

### 3.2 Red culture

Currently, no public data are available to measure the influence of red culture. Red culture originated during the Chinese Revolution and can be traced back to the founding of the Communist Party of China in 1921. This peaked when the People's Republic of China (PRC) was founded in 1949. After China implemented its reform and opening-up policy in 1978, the influence of red culture weakened slightly due to the continuous influx of foreign capital, talent, and culture. After China's reform and opening-up achieved remarkable results and its economy continued to grow rapidly for over 30 years, Chinese people have increasingly attached importance to red culture since the 1990s. The once silent “Red Classics” have gradually returned to the contemporary cultural life of Chinese people. In terms of film and television dramas, several red culture films and TV dramas have emerged [[41], [42], [43]]. An increasing number of corporations have begun to pay attention to Red Culture, education, and training. Enterprises engaged in red culture education and training have emerged and show sustained growth trends. Chinese law stipulates that corporations' business must be limited to the business scope registered with the State Administration for Market Regulation. Therefore, the greater the number of red cultural enterprises in a city per unit population, the stronger the supply and demand for red cultural education, training, and inheritance. The greater the impact of red culture on other enterprises in the city. Based on this argument, we constructed independent variables for the empirical tests.

Finally, this study collects all the information on red culture enterprises (enterprises whose business scope includes “red culture”) from qcc.com, whose data comes from the State Administration for Market Regulation, but the query is more convenient. According to the establishment dates and registration locations, red cultural enterprises in each Chinese city from 2008 to 2021 were counted. By the end of 2021, there were 4337 red culture enterprises. The number of red culture enterprises was divided by the city's total population to provide a proxy variable for the influence of red culture. Moreover, the number of red cultural enterprises is divided by the city's land area to provide another variable for robustness checks. The more red culture enterprises in a unit population or a unit land area of the city where a corporation is located, the greater the influence of red culture on the corporation.

### 3.3 Variables

Referring to existing literature, the independent, dependent, and control variables are listed in Table 1.

**Table 1:** Definition of variables.

Type	Variable	Symbol	Variable definition
Dependent variable	Corporates' cash holdings	*CS*	Cash and cash equivalents are divided by total assets
		*rCS*	*CS* minus industry average
Independent variables	Red culture influence	*RED*	The number of red culture enterprises is divided by total population of city
		*rRED*	The number of red culture enterprises is divided by the city land area
Control variables	Impact of the COVID-19	*Covid*	Dummy variable, value is 1 in 2020 and 2021, otherwise value is 0
	Corporate scale	*Size*	The natural logarithm of total assets
	Corporate age	*Age*	The natural logarithm is obtained by subtracting the year of corporate establishment from the current year plus one.
	Tobin Q value of the corporate	*MB*	The natural logarithm of the corporate's equity value plus the debt value is divided by the book value of total assets (to avoid negative numbers, the Tobin Q value is multiplied by 10 and then taken as a natural logarithm).
	Operating cash flow	*Cf*	The net cash flow from the operating activities of the corporate is divided by non-cash assets (total assets minus cash and cash equivalents)
	Dividend and interest payments	*PAY*	The cash paid by the corporate as dividend profits and interest is divided by operating income.
	The long-term investments	*Capx*	The cash paid by corporates to purchase fixed assets, intangible, and other long-term assets is divided by the average total assets.
	Corporate growth	*Grow*	The annual growth rate of business income.
	The size of the board of directors	*Bsize*	The natural logarithm of the number of directors.
	The proportion of independent directors	*Indep*	The number of independent directors is divided by the number of directors.
	Economic development level	*PGDP*	Per capita real GDP of the city.
	Financial development level	*FSIZE*	The city's loan balance is divided by the city's GDP

### 3.4 Data

#### 3.4.1 Data source

The 2008 global financial crisis changed China's financial system and had a far-reaching impact on corporations. This study conducts an empirical analysis based on data from Chinese A-share listed companies from 2008 to 2021. Owing to China's vast territory, its development is uneven. When Chinese scholars study the impact of macro variables on listed Chinese companies, city macro variables are often the first choice. Therefore, we use the number of red cultural enterprises in the city where the listed companies are registered for the macro variable of red cultural influence.

This study uses the following sample selection criteria: (1) eliminates all samples of financial companies, (2) removes company samples with fewer than two annual observations, and (3) excludes samples with missing data. After the above processing, 33,016 year-corporate observations were obtained. In addition, continuous variables were winsorized up and down by 1% to eliminate the influence of outliers.

The number of red culture enterprises was obtained from qcc.com, an enterprise credit query tool under Qichacha Technology Co., Ltd., whose data comes from the State Administration for Market Regulation. However, the query is more convenient. It provides a batch export function that can export all the query results in batches. It stores basic information about various types of Chinese enterprises. We use “red culture” as our focus keyword in qcc.com to search for all corporations with red culture in their business scope and then download the registration place, establishment time, and other information. After excluding enterprises with missing establishment times and those canceled by the end of 2021, the number of red cultural enterprises was 4337. The cities’ population, GDP, and loan balance were obtained from the China City Statistical Yearbook. The listed companies' information was obtained from the Wind database, whose data were obtained from the annual reports published by listed companies.

#### 3.4.2 Summary statistics

Table 2 reports the cash holdings and other variables of Chinese A-share listed companies from 2008 to 2021. The average cash holdings of the corporates is 16.3272%, the minimum is only 0.5309%, and the maximum is 71.1220%. There is a large gap between the two. After subtracting the industry average for industry adjustment, the average of corporates' cash holdings is negative, indicating that more corporates are below the industry average.

**Table 2:** Summary statistics.

Variables	Obs	Mean	Std.Dev.	Min	Max
*CS* (%)	33,015	16.3273	13.1169	0.5309	71.1220
*rCS* (%)	33,015	−0.2800	12.1742	−23.2597	46.5017
*RED* (red enterprises/per thousand persons)	33,015	0.0380	0.1031	0.0000	0.5787
*rRED* (red enterprises/per million square kilometers)	33,015	0.0494	0.1618	0.0000	0.9729
*Size* (log)	33,015	22.1752	1.3759	15.4177	30.3704
*Capx* (%)	33,015	5.1507	5.4877	−3.7500	28.4204
*Grow* (%)	33,015	17.7274	43.4623	−60.8480	296.0220
*Cf* (%)	33,015	6.6640	11.4301	−24.8024	80.9070
*Age* (log)	33,015	2.9034	0.3363	0.0000	4.2047
*PAY* (%)	33,015	5.4720	6.4600	0.0000	44.2492
*MB* (log)	33,015	3.0533	0.5911	1.4882	8.2764
*Bsize* (log)	33,015	2.1383	0.2015	1.0986	2.9957
*Indep* (%)	33,015	37.3494	5.4863	0.0000	80.0000
*PGDP* (thousand yuan/person)	33,015	13.1902	9.9332	1.4001	44.2841
*FSIZE* (%)	33,015	157.3472	66.1405	39.8837	373.7555

### 3.5 Empirical model

This study uses the following model to test the research hypothesis:

(1) $${CS}_{i,t}=\alpha_{0}+\beta_{1}RED_{i,t}+\beta_{2}X+\alpha_{ik}+\alpha_{ik}\times Covid+Covid+\lambda_{t}+\varepsilon_{i,t}$$

where *i*, *t*, and *k* are corporate, year, and industry subscripts, respectively; $\alpha_{ik}$ captures the industry effects; $\alpha_{ik}\times Covid$ captures the impact effects of the COVID-19 on different industries; $\lambda_{t}$ captures the year effects, and $\varepsilon_{i,t}$ is the error term.

${CS}_{i,t}$ is the dependent variable for corporate cash holdings. $RED_{i,t}$ is the independent variable; that is, the influence of red culture on corporations. $\beta_{1}$ is the coefficient of the independent variable. If it is significantly negative, then a red culture is conducive to reducing corporate cash holdings. ***X*** is a control variable. Following the existing literature [34,37,44,45], this study controls for corporate scale, corporate age, Tobin's Q value of the corporation, operating cash flow, dividends and interest payments, long-term investments, corporate growth, size of the board of directors, and the proportion of independent directors. Moreover, to control for endogenicity, this study also controls for two macroscopic variables: economic and financial development levels. Tobin's Q value of the corporation is the equity market value plus the debt book value divided by the total asset book value, and these values are at the end of the fiscal year.

## 4 Results

Section 2 theorizes that red culture can reduce corporate cash holdings. In Section 3, we design the proxy variable and empirical model equation. This section presents our empirical results.

### 4.1 Correlation matrix

Table 3 reports the correlation matrix of variables. The table shows that the correlation coefficient of *CS* and *RED* is −0.028, and the p-value is less than 0.001. They are significantly negatively correlated at the 1% significance level.

**Table 3:** Correlation matrix of variables.

Variables	(1)	(2)	(3)	(4)	(5)	(6)	(7)	(8)	(9)	(10)	(11)	(12)	(13)
(1) *CS*	1.000												
(2) *RED*	−0.028	1.000											
	(0.000)												
(3) *Size*	−0.245	0.027	1.000										
	(0.000)	(0.000)											
(4) *Capx*	−0.021	−0.009	−0.010	1.000									
	(0.009)	(1.000)	(0.996)										
(5) *Grow*	0.028	−0.006	0.041	0.105	1.000								
	(0.000)	(1.000)	(0.000)	(0.000)									
(6) Cf	0.329	−0.008	0.019	0.134	0.045	1.000							
	(0.000)	(1.000)	(0.051)	(0.000)	(0.000)								
(7) *Age*	−0.167	0.111	0.159	−0.201	−0.040	−0.038	1.000						
	(0.000)	(0.000)	(0.000)	(0.000)	(0.000)	(0.000)							
(8) *PAY*	−0.063	−0.003	0.177	−0.008	−0.110	0.029	0.050	1.000					
	(0.000)	(1.000)	(0.000)	(1.000)	(0.000)	(0.000)	(0.000)						
(9) *MB*	0.299	0.008	−0.564	0.063	0.090	0.155	−0.129	−0.105	1.000				
	(0.000)	(1.000)	(0.000)	(0.000)	(0.000)	(0.000)	(0.000)	(0.000)					
(10) *Bsize*	−0.038	−0.048	0.258	0.036	−0.006	0.033	−0.012	0.073	−0.177	1.000			
	(0.000)	(0.000)	(0.000)	(0.000)	(1.000)	(0.000)	(0.940)	(0.000)	(0.000)				
(11) *Indep*	0.003	0.050	0.019	−0.014	−0.003	−0.017	−0.009	−0.012	0.040	−0.501	1.000		
	(1.000)	(0.000)	(0.043)	(0.620)	(1.000)	(0.138)	(1.000)	(0.884)	(0.000)	(0.000)			
(12) *PGDP*	0.051	0.285	0.038	−0.051	0.007	−0.015	0.160	−0.005	0.045	−0.109	0.081	1.000	
	(0.000)	(0.000)	(0.000)	(0.000)	(1.000)	(0.381)	(0.000)	(1.000)	(0.000)	(0.000)	(0.000)		
(13) *FSIZE*	0.018	0.113	0.126	−0.120	−0.015	−0.028	0.178	0.029	−0.024	−0.061	0.064	0.249	1.000
	(0.092)	(0.000)	(0.000)	(0.000)	(0.459)	(0.000)	(0.000)	(0.000)	(0.001)	(0.000)	(0.000)	(0.000)	

Notes: p-values in parentheses.

### 4.2 Baseline regression

For panel data with large N and small T (N refers to the number of individuals, and T refers to the number of periods), the unit root and cointegration tests are not required [46,47]. Because the number of periods of the panel data analyzed in this study was small (T = 14) and the number of individuals was large (N = 3613), the panel unit root and cointegration tests were not required. We estimate Eq. (1); the results are presented in Table 4.

**Table 4:** Baseline regress results.

	(1)	(2)	(3)	(4)	(5)
Variables	*CS*	*CS*	*CS*	*CS*	*CS*
*RED*	−7.7830**	−1.9938***	−1.4095***	−1.4082***	−1.5979***
	(3.5191)	(0.3109)	(0.2832)	(0.2864)	(0.3311)
*Ind_A*×*Covid*	−2.1800	−1.2441	−0.5662	−0.5034	−0.6230
	(1.5750)	(1.6639)	(1.8950)	(1.9016)	(1.8458)
*Ind_B*×*Covid*	−0.5644	0.8019	0.3393	0.4117	0.2167
	(1.6528)	(1.8069)	(1.7901)	(1.7951)	(1.7568)
*Ind_C*×*Covid*	−2.4315	−2.1316	−2.0238	−1.9528	−2.1398
	(1.5209)	(1.4911)	(1.8077)	(1.8030)	(1.7717)
*Ind_D*×*Covid*	−1.4479	−0.7492	−0.0954	−0.0334	−0.3481
	(1.4017)	(1.6357)	(1.8605)	(1.8667)	(1.8287)
*Ind_E*×*Covid*	−3.0572**	−1.9132	−1.7353	−1.6551	−1.6568
	(1.2074)	(1.2004)	(1.2976)	(1.3086)	(1.3004)
*Ind_F*×*Covid*	−2.0665	−1.1350	−1.0586	−1.0064	−1.1539
	(1.3949)	(1.4577)	(1.5848)	(1.5928)	(1.5660)
*Ind_G*×*Covid*	−3.7803	−1.5673	−1.2991	−1.2425	−1.5399
	(2.9955)	(2.5317)	(2.5488)	(2.5275)	(2.5176)
*Ind_H*×*Covid*	−4.8961*	−2.0280	−0.8999	−0.8580	−0.9823
	(2.8354)	(2.4643)	(2.0910)	(2.1084)	(2.0920)
*Ind_I*×*Covid*	−4.5282**	−5.1026**	−4.6338*	−4.5393*	−4.5266*
	(2.2480)	(2.1943)	(2.4667)	(2.4674)	(2.4463)
*Ind_K*×*Covid*	−1.8787	−0.9111	−0.9655	−0.9049	−1.0583
	(1.4513)	(1.3671)	(1.3227)	(1.3319)	(1.3390)
*Ind_L*×*Covid*	−2.8491	−3.0237	−2.2454	−2.1676	−2.1254
	(1.9288)	(2.0737)	(2.2249)	(2.2142)	(2.2072)
*Ind_M*×*Covid*	−2.3635	−1.5025	−1.8574	−1.8055	−2.0354
	(2.4290)	(2.4637)	(2.6443)	(2.6531)	(2.6251)
*Ind_N*×*Covid*	−3.1355**	−3.5331**	−3.1142*	−3.0279*	−3.1109*
	(1.5084)	(1.7278)	(1.7579)	(1.7655)	(1.7425)
*Ind_O*×*Covid*	−16.4940***	−19.4894***	−20.3734***	−20.5737***	−20.2920***
	(4.1370)	(4.1172)	(4.0958)	(4.2187)	(4.3025)
*Ind_P*×*Covid*	−2.0188	−4.0980	−2.4746	−2.3841	−2.2320
	(2.7866)	(2.7060)	(2.5795)	(2.5575)	(2.4722)
*Ind_Q*×*Covid*	0.7891	−0.5960	−0.5514	−0.4082	−0.5683
	(1.8137)	(1.6223)	(1.9452)	(1.9649)	(1.9703)
*Ind_R*×*Covid*	−2.6430	−2.8260**	−2.8184**	−2.7779**	−3.0114**
	(1.6807)	(1.2011)	(1.2389)	(1.2451)	(1.2532)
*Covid*	0.7593	5.1412**	5.2821**	5.3969**	4.1919**
	(1.3079)	(2.0872)	(2.3058)	(2.2915)	(2.0701)
*Size*		−1.2134***	−1.3193***	−1.3716***	−1.3905***
		(0.3799)	(0.3936)	(0.3932)	(0.3922)
*Age*		−7.6571***	−7.4608***	−7.4933***	−7.4360***
		(2.0283)	(1.9961)	(1.9880)	(2.0042)
*MB*		3.8607***	2.8672***	2.8591***	2.8016***
		(0.5800)	(0.5908)	(0.5935)	(0.5891)
Cf			0.2746***	0.2745***	0.2750***
			(0.0094)	(0.0094)	(0.0092)
*PAY*			0.0050	0.0053	0.0051
			(0.0226)	(0.0227)	(0.0227)
*Capx*			−0.0683***	−0.0685***	−0.0679***
			(0.0153)	(0.0153)	(0.0159)
*Grow*			0.0001	0.0001	0.0000
			(0.0021)	(0.0021)	(0.0021)
*Bsize*				1.3508**	1.4676**
				(0.6217)	(0.6217)
*Indep*				0.0057	0.0040
				(0.0185)	(0.0184)
*PGDP*					0.0830***
					(0.0262)
*FSIZE*					0.0109***
					(0.0022)
*Constant*	14.6852***	46.9066***	50.3214***	48.3846***	46.8732***
	(2.1699)	(13.4984)	(13.6972)	(14.2815)	(13.9562)
Industry FE	YES	YES	YES	YES	YES
Year FE	YES	YES	YES	YES	YES
Observations	33,015	33,015	33,015	33,015	33,015
R-squared	0.0332	0.1454	0.2023	0.2025	0.2026
N	3613	3613	3613	3613	3613

Note: Standard deviations are in parentheses. *,**, and *** denote statistical significance at the 10%, 5%, and 1% level, respectively. According to the China Securities Regulatory Commission classification, listed Chinese companies are divided into 19 industries (see Table 9 for details). We eliminate the financial industry (Ind_J); only 17 industry dummy variables are generated for the remaining 18 industries. A comprehensive industry was used as the benchmark for Sushima.

Column (1) shows the estimated results considering only the independent variables. This shows that the coefficient of the red culture influence (p < 0.05) is significantly negative. The empirical results support our hypothesis that a red culture can reduce corporate cash holdings. For each additional unit of red cultural influence, corporate cash holdings decrease by 7.7830% points.

The decreased cash-holding behavior of corporations may result from difficulties in corporate access to credit. As a matter of fact, in recent years, Chinese banks have increasingly faced liquidity problems. Moreover, corporations’ cash-holding behavior may also result from the macroeconomy. Thus, in Column (2), we control for levels of economic and financial development. The results show that the coefficient of the influence of red culture (p-value < 0.01) is significantly negative and that red culture can reduce corporate cash holdings.

Banks dominate China's financial system. The older the corporation, the more fully the bank understands it and the stronger its debt financing ability, weakening its preventive motivation to hold cash [37]. The larger the corporation, the stronger its anti-risk ability, the easier it is to obtain more debt funds [48], the weaker its preventive motivation to hold cash, and the more likely it is to reduce its cash holdings [2]. A corporation's Tobin's Q can represent investment opportunities within a certain period [49,50]. The greater the investment opportunities, the more cash corporate reserves capture them [2]. To this end, in Column (3), we add three control variables: corporate size, corporate age, and the natural logarithm of Tobin's Q. After controlling for these three variables, the coefficient of red culture's influence (p-value < 0.01) is significantly negative; therefore, red culture can reduce corporate cash holdings.

The more abundant the operating cash flow of a corporation, the more it will need to invest [1], and the more likely it is to reserve funds for investment. Therefore, the more abundant the operating cash, the higher the corporate cash holdings. Paying dividends to shareholders and interest in creditors on the schedule is conducive to establishing a good corporate image, thereby increasing opportunities for external cooperation, which is conducive to corporate investment [1]. This also encourages corporations to reserve cash for investments. Long-term investments typically consume substantial resources. The more long-term the investments, the lower the cash holdings [2]. The higher the growth, the more positive the managers' expectations for the future and the more likely it is to reduce the preventive motivation of cash holding; hence, the lower the corporate cash holding [37]. Column (4) controls for operating cash flows, dividends, interest payments, long-term investments, and corporate growth. The results show that the coefficient of the influence of red culture (p-value < 0.01) is significantly negative and that red culture can reduce corporate cash holdings.

The larger the board of directors, the more difficult it is to reach a consensus on risky project investments [51], and corporations may be forced to retain cash. China's independent directors usually lack sufficient understanding of the operation of the corporate and have weak supervision over its management, thus increasing the cash holdings of the corporate. In Column (5), we control for the size of the board of directors and the proportion of independent directors. The results show that the coefficient of the influence of red culture (p < 0.01) is significantly negative; hence, red culture can reduce corporate cash holdings. After adding all the control variables, corporations' cash holdings decrease by 1.5979% points for each unit of red cultural influence.

In estimating Eq. (1), we control for COVID-19(*Covid*), the interaction between COVID-19 and industries (*Ind_A* × *Covid* ∼ *Ind_R* × *Covid*). In Column (5), the coefficient of *Covid* is significantly positive (p < 0.05); Among the interaction items between *Covid* and industries (*Ind_A* × *Covid* *∼* *Ind_R* × *Covid*), the coefficient of the interaction items between *Covid* and Information transmission, software, and information technology services industry (*Ind_I* × *Covid*) is significantly negative (p < 0.10), the coefficient of the interaction items between *Covid* and “Water conservancy, environment, and public facilities management industry” (*Ind_N* × *Covid*) is significantly negative (p < 0.10), and the coefficient of the interaction items between *Covid* and “Residential services, repair, and other services industry” (*Ind_O* × *Covid*) is significantly negative (<0.01), the coefficient of interaction between the *Covid* and “Culture, sports, and entertainment industry” (*Ind_R* × *Covid*) is significantly negative (p < 0.05). The interaction between *Covid* and other industries was not significant. This indicates that the COVID-19 pandemic has generally increased corporate cash holdings. According to coefficients of *Covid*, the interaction between *Covid* and the industries, COVID-19 finally reduced their cash holdings for corporates in the “Information transmission, software, and information technology services industry”, and “Residential services, repair, and other services industry.” For corporates in “Water conservancy, environment, and public facilities management industry,” “Culture, sports, and entertainment industry,” COVID-19 has increased their cash holdings. Still, it is not as large as corporates in other industries.

**Fig. 2:**
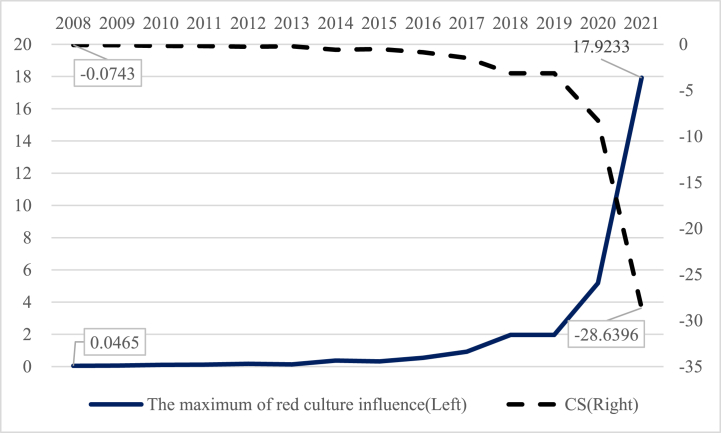
The maximum of red culture influence and its reducing effect on corporate cash holdings from 2008 to 2021.

**Fig. 3:**
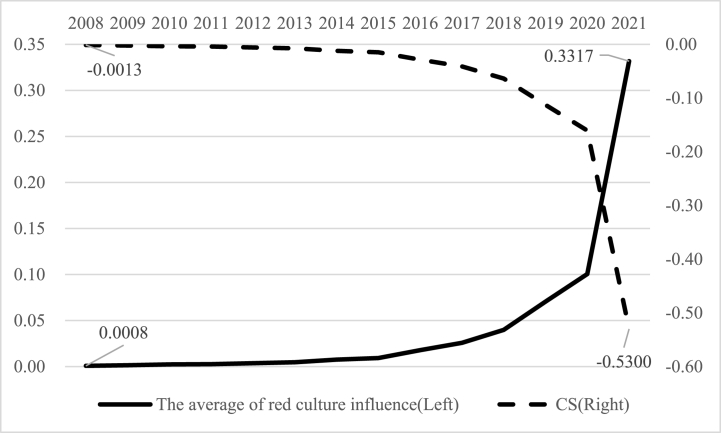
The average of red culture influence and its reducing effect on corporate cash holdings from 2008 to 2021.

According to Column (5), regardless of whether the average or the maximum red culture influence has increased yearly from 2008 to 2021, corporations' cash holdings have decreased year by year, and the decline rate is more significant after 2014 (Figs. 2 and 3).

### 4.3 Robustness checks

In Table 4, when the control variables are gradually added, the influence of red culture becomes significant, which can be regarded as a robustness test. We further check robustness through endogenous treatment, changing the measurement of corporate cash holdings and red culture influence, changing the estimation model, and controlling for R&D investments.

#### 4.3.1 Endogenous treatment

As a micro variable, corporate cash holdings hardly affect the influence of red culture, which is a macro variable. Therefore, it is difficult to establish a two-way causal relationship between corporate cash holdings and the influence of red culture. However, there may be endogenous problems due to measurement errors caused by the influence of red blood culture.

Therefore, we first use the individual fixed effects model to estimate Eq. (1) to alleviate endogeneity; the results are shown in Column (1) of Table 5. Second, following Faccio, Marchica, and Mura (2011) [52], Laeven and Levine (2007, 2009) [53,54], and Chen, Yan, and Chen (2021) [55], this study takes the average number of red cultural influences in other cities in the same year as the instrumental variable (*ivRED*), corporate cash holdings as the dependent variable, and red cultural influence as the independent variable. We then use the instrumental variable method and control for individual fixed effects to re-estimate Eq. (1). The *Cragg Donald Wald F statistic* and *Kleibergen-Paap rk Wald F statistic* of the weak instrumental variable test were 1920.850 and 178.168, respectively, far greater than the critical value of 16.38 under a 10% bias. This passed the weak intravenous test. The Hausman test was performed. The chi-square statistic was 416.46, and the p-value was <0.0001. Therefore, the influence of red blood culture was endogenous.

**Table 5:** Estimated results of robustness checks.

	(1)	(2)	(3)	(4)	(5)	(6)
Variables	*CS*	*CS*	*rCS*	*CS*	*CS*	*CS*
*RED*	−3.7518**	−8.4712***	−1.7100***		−2.4994***	−1.6754***
	(1.5039)	(2.8113)	(0.3975)		(0.9671)	(0.3188)
*rRED*				−2.1153***		
				(0.5544)		
*RD*						0.2303**
						(0.0965)
Individual FE	YES	YES	NO	NO	NO	NO
Year FE	YES	YES	YES	YES	YES	YES
Control variables	YES	YES	YES	YES	YES	YES
Industry FE	NO	NO	YES	YES	YES	YES
Industry × Covid	NO	NO	YES	YES	YES	YES
Observations	33,015	33,015	33,015	33,015	26,363	33,015
R-squared	0.2053	0.2046	0.1120	0.2033	0.2135	0.2020
N	3613	3613	3613	3508	3613	3613

Note: Standard deviations are in parentheses. *,**, and *** denote statistical significance at the 10%, 5%, and 1% level, respectively.

*ivRED* as the instrumental variable, xtivreg2 was used to estimate Eq. (1) with the GMM option; the results are shown in Column (2) of Table 5. In Columns (1) and (2), the influence of red culture was significantly negative at the 1% level. Therefore, even after excluding endogeneity, red culture reduces corporate cash holdings.

#### 4.3.2 Changing the measurement of corporates’ cash holdings

Industry differences exist in corporate cash holdings. Florackis and Sainani (2018) [37] make industry adjustments for corporate cash holdings in their study. We refer to Chinese studies on the cash holdings of Chinese listed companies and obtain *rCS* after the industry adjustment of corporate cash holdings. Then, we re-estimate Eq. (1), and the results are shown in Column (3) of Table 5. The influence of the red culture was significantly negative at the 1% level. Therefore, even after changing the measurement method for corporate cash holdings, the influence of red culture could still reduce corporate cash holdings.

#### 4.3.3 Remeasuring the influence of red culture

The number of red cultural enterprises within the unit land area of the corporation's location reflects the market demand for red culture on the demand side and the impact of red culture on the supply side. Therefore, the number of red culture enterprises per unit land area of the city where the corporation is located can also reflect the influence of red culture on the corporation. Therefore, this study takes the number of red culture enterprises divided by the city land area as another proxy variable for the influence of red culture and re-estimates Eq. (1). The results are shown in Column (4) of Table 5. The influence of the red culture was significantly negative at the 1% level. Changing the measurement of the influence of red culture reduces corporate cash holdings. Thus, the research hypotheses were confirmed.

#### 4.3.4 Eliminating interpolated data

Due to data availability, we interpolated the city population, city loan balance, and city GDP for 2020 and 2021. Here, we exclude the interpolated data and reestimate Eq. (1). The results are shown in Column (5) of Table 5. The influence of the red culture was significantly negative at the 1% level. Thus, even after eliminating interpolated data, the influence of red culture can still reduce corporate cash holdings.

#### 4.3.5 Controlling for R&D investment

Corporate R&D investment is resource intensive. To invest in R&D, enterprises must reserve cash and other resources to increase their cash holdings [34]. Global cultures can be divided into collectivism and individualism [56]. Individualism encourages innovation and R&D investment. The Chinese culture is collectivist. The continuous decline in the cash holdings of Chinese listed companies may be caused by their low R&D investment of Chinese listed companies. Therefore, we divide R&D investment by total assets to obtain *Rd*. We control for *RD* and reestimate Eq. (1), and the results are shown in Column (6) of Table 5. The influence of the red culture was significantly negative at the 1% level. Therefore, even after controlling for R&D investment, the influence of red culture can still reduce corporate cash holdings.

In summary, red culture reduces corporate cash holdings, even after excluding endogeneity, changing the measurement of corporate cash holdings and the influence of red culture, eliminating interpolated data, and controlling for corporate R&D investment.

## 5 Heterogeneity analysis

### 5.1 Long-term investment heterogeneity

In 2008, the long-term investments of listed Chinese enterprises accounted for 6.93% of average total assets, which decreased to 4.71% by 2021. Since 2008, the long-term investments of listed Chinese companies have generally shown a downward trend, decreasing by 2.22% over the past 13 years. A possible reason is that since 2008, Chinese macroeconomic uncertainty has increased, and corporations’ future expectations have been poor, which has weakened the long-term development motivation of corporations and reduced long-term investment.

Corporations' long-term investments consume large amounts of resources. The longer-term the investment, the more cash resources are consumed, and the lower the corporations’ cash holdings [2]. Red culture can inculcate psychological ownership among managers. Individuals can improve their control over and protect target objects by being driven by psychological ownership. Managers with psychological ownership towards the corporation actively contribute to the corporation and invest time and effort in improving it [25]. Psychological ownership encourages managers to engage in out-of-role behaviors, improve work efficiency and performance, and develop a competitive advantage. Improving corporations and enhancing their competitive advantages are conducive to enhancing their anti-risk ability and profitability, which in turn improve corporate debt financing ability. Improvement in debt-financing ability means corporations can raise more external funds for long-term investments. They can also consume the cash generated during operational activities, thus reducing corporate cash holdings. Therefore, we believe that the role of red culture in reducing corporate cash holdings has long-term investment heterogeneity and that the role of red culture in corporations with high long-term investments will be greater than that in corporations with low long-term investments.

Here, we measure corporations' long-term investments by subtracting the cash paid by the corporation to purchase fixed assets, intangible assets, and other long-term assets from the cash received by the corporations for disposing of fixed assets, intangible assets, and other long-term assets, and then dividing it by the average total assets. Based on Florackis and Sainani's (2018) [37] model of dividing types by annual median, we classify firms with long-term investments above the median yearly as high-term investment corporations and vice versa. Then, with *CS* as the dependent variable and the influence of red culture as the independent variable, the variable coefficient fixed-effects model was used to estimate Eq. (1), and the result is shown in Column (1) of Table 6, where the dependent variable is replaced by *rCS*, and the variable coefficient fixed-effect model is used to estimate Eq. (1). The results are shown in Column (2) of Table 6. We replaced the independent variable with *rRED* and re-estimated Eq. (1) in the above order; the results are shown in Columns (3) and (4) of Table 6.

**Table 6:** Estimated results of long-term investment heterogeneity.

	(1)	(2)	(3)	(4)
Variables	*CS*	*rCS*	*CS*	*rCS*
*RED* (Low)	−0.5177	−0.5026		
	(0.6441)	(0.7172)		
*RED* (High)	−2.5739***	−2.8043***		
	(0.4432)	(0.4396)		
*rRED* (Low)			−1.6045***	−1.4031***
			(0.5047)	(0.3430)
*rRED* (High)			−2.5887***	−2.5969***
			(0.6907)	(0.6027)
Industry FE	YES	YES	YES	YES
Industry × Covid	YES	YES	YES	YES
Year FE	YES	YES	YES	YES
Control variables	YES	YES	YES	YES
Observations	33,016	33,016	33,016	33,016
R-squared	0.2027	0.1121	0.2033	0.1127
N	3613	3613	3613	3613

Notes: This table reports the estimated results for long-term investment heterogeneity. The standard deviations are shown in parentheses. *, **, and *** denote statistical significance at the 10%, 5%, and 1% level, respectively.

From Table 6, the following can be seen. First, the influence of red culture is partially negative in low long-term investment enterprises. Second, the absolute value of the red cultural influence coefficient of corporations with high long-term investments is greater than that of corporations with low long-term investments. The empirical results confirm our conjecture that the role of red culture in reducing corporate cash holdings has long-term investment heterogeneity; that is, the role of red culture in high long-term investment corporations is greater than that in low long-term investment corporations.

### 5.2 Enterprise investment opportunity heterogeneity

According to Tobin's theory, whether a corporation continues to invest depends on its marginal Tobin's Q value. Corporations can continue to invest in projects with positive net present values until the marginal Tobin's Q is zero. Therefore, Tobin's Q of a corporation also indicates its investment opportunity [49,50]. When other conditions remain unchanged, the higher Tobin's Q, the greater the investment opportunity. In 2008, the average investment opportunity of Chinese listed companies was 1.79 times; that is, the market value of total assets was 1.79 times the book value, which rose to 2.51 times by 2021, an increase of 0.72 times in 13 years. Generally, there is an upward trend with fluctuations.

A long-term investment is the net investment in fixed, intangible, and other long-term assets. Corporations have different investment opportunities, and they determine their futures. Therefore, to seize future investment opportunities, corporations hold more cash out of a prevention motivation [2]. The higher a corporation's investment opportunity, the more cash it holds. When individuals have psychological ownership towards the organization, they recognize the organization as “theirs” [25], regard themselves as the organization's owners, and pay attention to the effective realization of the organization's long-term development. Therefore, when managers have psychological ownership of a corporation, they invest more in fixed assets, thereby increasing the proportion of fixed assets. The higher the investment opportunity of a corporation, the more fixed asset investment it can make and the more cash it can consume to seize the investment opportunity. Thus, the role of red culture in reducing corporate cash holdings is more evident in corporations with high investment opportunities. In addition, corporations with high investment opportunities have better corporate value and are more likely to be favored by creditors such as banks, making it easier to obtain debt support. This also indicates that the role of red culture in reducing corporate cash holdings is more obvious in corporations with high investment opportunities. Therefore, we believe that the role of red culture in reducing corporate cash holdings is heterogeneous in investment opportunities and that the role of red culture in corporations with high investment opportunities will be greater than that in corporations with low investment opportunities.

Here, we measure corporate investment opportunities by dividing the market value of total corporate assets by book value. Drawing on Florackis and Sainani's (2018) [2] model of classifying the two types based on the annual median, we classify corporations with annual investment opportunities above the median as corporations with high investment opportunities and vice versa. Next, with *CS* as the dependent variable and the influence of the red culture as the independent variable, the variable coefficient fixed-effects model was used to estimate Eq. (1). The results are shown in Column (1) of Table 7. The dependent variable was replaced with *rCS*, and the variable coefficient fixed-effect model was used to estimate Eq. (1). The results are shown in Column (2) of Table 7. We replaced the independent variable with *rRED* and re-estimated Eq. (1) in the above order; the results are shown in Columns (3) and (4) of Table 7.

**Table 7:** Estimated results of investment opportunity heterogeneity.

	(1)	(2)	(3)	(4)
Variables	*CS*	*rCS*	*CS*	*rCS*
*RED* (Low)	−0.1651	−0.9709		
	(0.9783)	(0.8359)		
*RED* (High)	−3.0408***	−2.4596***		
	(0.8616)	(0.6067)		
*rRED* (Low)			−1.0793**	−1.3555***
			(0.4722)	(0.3817)
*rRED* (High)			−3.0968***	−2.6581***
			(0.9225)	(0.6664)
Industry FE	YES	YES	YES	YES
Industry × Covid	YES	YES	YES	YES
Year FE	YES	YES	YES	YES
Control variables	YES	YES	YES	YES
Observations	33,015	33,015	33,015	33,015
R-squared	0.2028	0.1121	0.2036	0.1128
N	3613	3613	3613	3613

Note: Standard deviations are in parentheses. *, **, and *** denote statistical significance at the 10%, 5%, and 1% level, respectively.

From Table 7, we can deduce the following. First, the influence of red culture is partially negative in enterprises with low investment opportunities. Second, the absolute value of the red culture influence coefficient for corporations with high investment opportunities is greater than that for corporations with low investment opportunities. Therefore, the empirical results confirm our conjecture that the role of red culture in reducing corporate cash holdings has the heterogeneity of investment opportunities, and the role of red culture in high-investment opportunity corporations is more significant than that in low-investment opportunity corporations.

### 5.3 Duality heterogeneity

In Europe, the United States, and other countries, separating the board chairperson and CEO has become a trend [57,58]. In some countries, laws stipulate that the chairperson and CEO must be separated [57]. In 2008, duality accounted for 14.61% of China's listed companies. In 2021, this proportion will increase to 29.51%, an increase of 14.89% over the past 13 years. Since 2008, the phenomenon of the board chairperson concurrently serving as CEO in Chinese listed companies has become increasingly evident, contrary to the trend in Europe, the United States, and other countries. Thus, in the two types of corporations, separated or merged, is there heterogeneity in the role of red culture in reducing corporate cash holdings?

We posit that red culture significantly reduces corporate cash holdings in Chinese-listed companies where the board chairperson is also the CEO. There are advantages and disadvantages to concurrently serving as the chairperson and CEO [58]. In Section 5.1, it was found that red culture can improve the debt financing ability of corporations, the board of directors is responsible for making decisions on the financing plan, and the board of directors can better understand the production and operation of the corporation if the chairperson concurrently serves as CEO [58]. Therefore, when the chairperson of the board of directors concurrently serves as the CEO, the board of directors can make more informed decisions on the financing plan, further playing the role of a red culture in improving the debt financing ability of corporations and reducing cash holdings. The chairperson concurrently serving as CEO can improve the freedom of corporate innovation, the efficiency of information communication, and the decision-making efficiency of the corporation [58,59], thereby improving corporate performance. When red culture urges the CEO to generate psychological ownership, the role of the chairperson as a CEO in improving corporate performance becomes more prominent. This will also improve corporations’ debt-financing capacity. Therefore, in corporations where the board chairperson concurrently serves as CEO, the role of red culture in reducing corporate cash holdings is greater.

We use the variable coefficient fixed-effect model to estimate Eq. (1), considering whether the board chairperson concurrently serves as CEO as the classification variable, *CS* as the dependent variable, and the influence of red culture as the independent variable. The results are shown in Column (1) of Table 8. The dependent variable was replaced with *rCS*, and the variable coefficient fixed-effect model was used to estimate Eq. (1). The results are shown in Column (2) of Table 8. We replaced the independent variable with *rRED* and re-estimated Eq. (1) in the above order, and the results are shown in Columns (3) and (4) of Table 8.

**Table 8:** Estimated results of duality heterogeneity.

	(1)	(2)	(3)	(4)
Variables	*CS*	*rCS*	*CS*	*rCS*
*RED* (NO)	−1.1776**	−1.3999**		
	(0.5277)	(0.5827)		
*RED* (YES)	−2.5902***	−2.4449***		
	(0.8035)	(0.5851)		
*rRED* (NO)			(0.0023)	(0.0015)
			−1.7542***	−1.7372***
*rRED* (YES)			(0.4039)	(0.3159)
			−2.7692***	−2.5398***
Industry FE	YES	YES	YES	YES
Industry × Covid	YES	YES	YES	YES
Year FE	YES	YES	YES	YES
Control variables	YES	YES	YES	YES
Observations	33,015	33,015	33,015	33,015
R-squared	0.2027	0.1120	0.2033	0.1127
N	3613	3613	3613	3613

Note: Standard deviations are in parentheses. *,**, and *** denote statistical significance at the 10%, 5%, and 1% level, respectively.

**Table 9:** Industry classification of China securities regulatory commission.

Number	Code	Name
1	A	Agriculture, forestry, animal husbandry, and fishery industry
2	B	Mining industry
3	C	Manufacturing industry
4	D	Electricity, heat, gas, and water production and supply industry
5	E	Construction industry
6	F	The wholesale and retail industry
7	G	Transportation, storage, and postal services industry
8	H	Accommodation and catering industry
9	I	Information transmission, software, and information technology services industry
10	J	Finance industry
11	K	Real estate industry
12	L	Leasing and business services industry
13	M	Scientific research and technology services industry
14	N	Water conservancy, environment, and public facilities management industry
15	O	Residential services, repair, and other services industry
16	P	Education industry
17	Q	Health and social work industry
18	R	Culture, sports, and entertainment industry
19	S	The comprehensive industry

From Table 8, we can deduce the following. First, the influence of red culture is partially negative in enterprises where the chairperson and CEO are separated. Second, the absolute influence coefficient of red culture in a corporation where the chairperson concurrently serves as CEO is greater than that in a corporation where the chairperson and CEO are separated. The empirical results confirm our conjecture that the role of red culture in reducing corporate cash holdings is dually heterogeneous, and the role of red culture in corporations with merged chairperson and CEO positions is greater than that in companies with separate chairperson and CEO positions.

## 6 Conclusions, recommendations, and limitations

### 6.1 Conclusions

This study examines the impact of red culture on the cash holdings of listed Chinese companies. We found that red culture can reduce corporate cash holdings. The greater the influence of red culture, the lower corporate cash holdings. This conclusion is still valid in the case of robustness checks such as using the instrumental variable method to control for endogeneity, changing the measurement method of the influence of red culture on corporate cash holdings, eliminating interpolated data, and considering R&D investments. The heterogeneity analysis shows that the role of red culture in reducing corporate cash holdings is heterogeneous: the role of red culture in high long-term investment corporations is greater than that in low long-term investment corporates; the role of red culture in high investment opportunity corporates is greater than that in low investment opportunity corporates; and the role of red culture in merged Chairperson and CEO position corporates is greater than that in separated chairperson and CEO position corporates.

### 6.2 Recommendations

The conclusions of this study have several recommendations:

First, red culture plays a role in reducing corporate cash holdings. To encourage corporations to reduce corporate cash holdings and improve corporate value, shareholders and board directors of corporations can start from the culture perspective and actively promote the core concept of red culture to help managers generate psychological ownership towards corporations and promote their ethical leadership.

Second, as noted, the role of red culture in reducing corporate cash holdings is heterogeneous; red culture plays a greater role in high long-term investment corporations than in low long-term investment corporates, in high investment opportunity corporates than in low investment opportunity corporates, and in duality corporations than in non-duality corporations. Chinese people have increasingly attached importance to red culture since the 1990s. Since 2014, the influence of red culture has significantly increased. Therefore, global investors can focus on three types of corporations: those with high long-term investment, those with high investment opportunities, and duality corporations.

### 6.3 Limitations

The topics discussed in this study are interdisciplinary. The study strictly follows the economic paradigm, proposes research hypotheses, develops empirical models, analyzes data resources, tests the research hypotheses, and draws conclusions. Therefore, this study refers to the methods adopted in the economic literature [34,37,44,45] to examine the impact of red culture on corporate cash holdings.

Based on existing literature, this study believes that red culture can help managers generate psychological ownership towards corporations and promote ethical leadership. Future research should empirically study the specific path through which red culture affects managers' psychological ownership towards corporations and their ethical leadership based on a sampling survey. This is a limitation of the present study.

## Author contribution statement

Zuohong Li: Analyzed and interpreted the data, contributed reagents, materials, and analysis tools, and wrote the paper.

Xiaohui Chen: Conceived and designed the experiments, performed the experiments, analyzed and interpreted the data, and wrote the manuscript.

Bo Yang: Analyzed and interpreted the data, contributed reagents, materials, and analysis tools, analyzed and interpreted the data, and wrote the manuscript.

## Data availability statement

Data will be made available on request.

## Declaration of interest's statement

The authors declare that they have no known competing financial interests or personal relationships that could have appeared to influence the work reported in this paper.
